# Gene Expression Microarray Data Meta-Analysis Identifies Candidate Genes and Molecular Mechanism Associated with Clear Cell Renal Cell Carcinoma 

**DOI:** 10.22074/cellj.2020.6561

**Published:** 2019-12-15

**Authors:** Ying Wang, Haibin Wei, Lizhi Song, Lu Xu, Jingyao Bao, Jiang Liu

**Affiliations:** 1 Institute of Aging Research, School of Medicine, Hangzhou Normal University, Hangzhou, Zhejiang, China; 2 Department of Pathology, Zhejiang Cancer Hospital, Hangzhou, Zhejiang, China

**Keywords:** Clear Cell Renal Cell Carcinoma, Immune Response, Protein-Protein Interaction Network

## Abstract

**Objective:**

We aimed to explore potential molecular mechanisms of clear cell renal cell carcinoma (ccRCC) and provide candidate target genes for ccRCC gene therapy.

**Materials and Methods:**

This is a bioinformatics-based study. Microarray datasets of GSE6344, GSE781 and GSE53000 were downloaded from Gene Expression Omnibus database. Using meta-analysis, differentially expressed genes (DEGs) were identified between ccRCC and normal samples, followed by Kyoto Encyclopedia of Genes and Genomes (KEGG) pathway and Gene Ontology (GO) function analyses. Then, protein-protein interaction (PPI) networks and modules were investigated. Furthermore, miRNAs-target gene regulatory network was constructed.

**Results:**

Total of 511 up-regulated and 444 down-regulated DEGs were determined in the present gene expression microarray data meta-analysis. These DEGs were enriched in functions like immune system process and pathways like Toll-like receptor signaling pathway. PPI network and eight modules were further constructed. A total of 10 outstanding DEGs including TYRO protein tyrosine kinase binding protein (*TYROBP*), interferon regulatory factor 7 (*IRF7*) and PPARG co-activator 1 alpha (*PPARGC1A*) were detected in PPI network. Furthermore, the miRNAs-target gene regulation analyses showed that miR-412 and miR-199b respectively targeted IRF7 and PPARGC1A to regulate the immune response in ccRCC.

**Conclusion:**

TYROBP, IRF7 and PPARGC1A might play important roles in ccRCC via taking part in the immune system process.

## Introduction

Clear cell renal cell carcinoma (ccRCC) is a type of RCC developed in adults (1). It has been reported that ccRCC is the most aggressive subtype of RCC (2). Surgery by radical or partial nephrectomy is the main choice for treatment of ccRCC. Although chemotherapy and immunotherapy could be applicable in patients with metastatic ccRCC, the outcome is unsatisfactory (3). Even some of ccRCC are along with the worst prognosis among the common epithelial tumors of the kidney (4). Therefore, exploring molecular biomarkers serving as diagnostic and therapeutic targets, when used alone or in combination with other clinical parameters, are urgently required for better clinical management.

Accumulating evidences suggest that certain differentially expressed genes (DEGs) are closely related to disease progression. Duns et al. (5) showed that histone methyltransferase gene SET domain containing 2 (*SETD2*) is a novel tumor suppressor gene in the process of ccRCC. A chromatin-remodeling gene ATrich interaction domain 1A (*ARID1A*) is considered to be a new prognostic marker in ccRCC (6). Actually, these genes often play important roles in ccRCC progression via certain function or specific pathways. A recent study indicated that frequent methylation of Kelch like ECH associated protein 1 (*KEAP1*) gene promoter was vital for ccRCC development via KEAP1/ nuclear factor erythroid-2 related factor (NRF2) pathway (7). Moreover, some microRNAs (miRNAs) are abnormally expressed in ccRCC and contribute to tumorigenesis. Urinary miRNAs have been proved to be the predictors of tumor metastasis in ccRCC (8). Yang et al. (9) showed that miR-506 was down-regulated in ccRCC and inhibited cell growth and metastasis via targeting flotillin 1. Although there have been many researches to find genetic biomarkers for ccRCC, characterization progress of the genetic events associated with this cancer is not fully clear yet.

In this study, meta-analysis was used to detect potential DEGs between ccRCC and normal samples based on three microarray datasets. Moreover, functional and pathway enrichment analyses were carried out for these DEGs. Then, protein-protein interaction (PPI) network was investigated. Furthermore, miRNA-target gene interaction network was constructed. We hoped to explore the underlying molecular mechanisms of ccRCC and provide candidate target genes for ccRCC gene therapy.

## Materials and Methods

### Microarray data and preprocessing

This is a bioinformatics-based study. Microarray datasets were downloaded from Gene Expression Omnibus database based on the data quantity, sample grouping, microarray platform (Affymetrix) and number of citation. Finally, three datasets were selected: GSE6344, GSE781 and GSE53000. The reasons for selection are as follows: i. Large data quantities, ii. Clear grouping of the experiment (tumor vs. normal), iii. Common microarray platforms (Affymetrix), iv. Consistent sample types (tissue samples). In details, 10 ccRCC and 10 normal tissue samples sequencing on the platform of GPL96 [HGU133A] Affymetrix Human Genome U133A Array were selected for analysis in microarray dataset GSE6344 (10). For GSE781 (11), 12 ccRCC and 5 normal tissue samples sequenced on the platform of GPL96 [HG-U133A] Affymetrix Human Genome U133A Array were selected. In addition, all samples in GSE53000 (12) (56 ccRCC and 6 normal tissue samples) sequencing on the platform of [HuGene-1_0-st] Affymetrix Human Gene 1.0 ST Array [transcript (gene) version] were used for analysis. Specially, GSE53000 included two samples of lymph node metastasis and one sample of venous thrombus metastasis. Thus, principal component analysis (PCA) was carried out for the 56 ccRCC tissue samples and 6 normal tissue samples. As shown in Figure S1 (Supplementary Online Information at www.celljournal.org), two samples from lymph node metastasis and one sample from venous thrombus metastasis were obviously clustered together, while separated from normal samples. So they could be unified as tumor group for the following analysis. Sample information of the three datasets was shown in Tables S1-S3 (Supplementary Online Information at www. celljournal.org). In order to eliminate the expression value heterogeneity of each gene in different platforms, CEL format of files of three datasets were pooled together and preprocessed using Affy software, including background adjustment, quantiles normalization, summarization and log2 fold change (FC) transformation using Robust Multi-array Average (RMA) algorithm in Affy package (13). The probe identities were converted to gene symbols based on the annotation files downloaded from different platforms. Probes that did not correspond to gene symbols were discarded. For different probes matched to one gene, the average value of different probes was used as the final expression value.

### Differentially expressed genes identification

DEGs of ccRCC and normal samples were separately screened based on multiple experimental datasets using MetaDE package in R software (14). The heterogeneity test was performed according to the expression values of each gene under different experimental platforms with the statistical parameters of tau2, Qvalue and Qpval. The tau2=0 [estimated amount of (residual) heterogeneity] and Qpval>0.05 (P values for the test of heterogeneity) represented significant homogeneity. Finally, BenjaminiHochber adjusted P value (fdr) <0.05, tau2=0 and Qpval >0.05 were considered as the cut-off criteria for DEGs selection. Furthermore, the log2 FC of ccRCC vs. normal >0 represented up-regulated DEGs, while log2 FC of ccRCC vs. normal <0 represented that DEGs were downregulated.

### Functional annotation and pathway enrichment analysis of differentially expressed genes

The clusterProfiler is an online tool applied for enrichment analysis (15). Gene Ontology (GO) functional annotation was used to analyze functions assembled with the up- and down-regulated genes by clusterProfiler. GO functions include molecular function (MF), biological process (BP) and cellular component (CC). To better understand pathways of the involved DEGs, Kyoto Encyclopedia of Genes and Genomes (KEGG) pathway enrichment analysis was performed using clusterProfiler. A P value (the significance threshold of the hypergeometric test) of <0.05 and count (the number of enriched genes) of >2 were used as the cut-off criteria for this analysis.

### Constructing protein-protein interaction network and modules analyses

PPI plays a key role in the completion of cellular functions, while they are usually correlated to each other in the form of a PPI network. The Search Tool for the Retrieval of Interacting Genes/Proteins (STRING) is a biological database of predicted and known PPIs. According to this database, the PPI network of DEG-encoding proteins in each group was constructed with the criterion of combined score (medium confidence) >0.4, and it was then visualized by the Cytoscape (version 3.2.0) software (National Institute of General Medical Sciences, USA). Score of nodes in the current network was analyzed using degree centrality, a topology property index. Higher node score presented more important node in the network, suggesting that is more likely the hub node in this network. Furthermore, the MCODE tool (National Institute of General Medical Sciences, USA) (16) in Cytoscape was used to screen the modules from the network.

### miRNA-target gene regulatory network construction

The potential ccRCC related miRNAs were explored based on Enrichr database. The miRNA-target gene regulatory network was constructed with the miRNA associating with up- and down-regulated gene based on Cytoscape software (National Institute of General Medical Sciences, USA).

## Results

### Differentially expressed genes investigation between clear cell renal cell carcinoma and control groups

With fdr <0.05, tau2=0 and Qpval >0.05, 955 DEGs were identified in ccRCC group compared to that of the normal controls, including 511 up-regulated and 444 down-regulated genes.

### Gene ontology function and Kyoto Encyclopedia of Genes and Genomes pathway enrichment analyses

**Fig 1:**
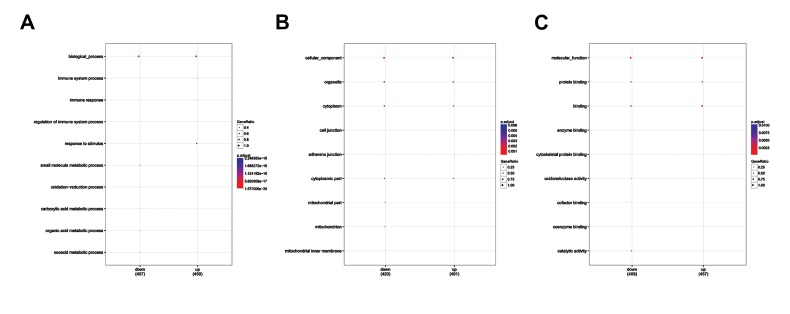
GO functional enrichment analysis for DEGs between ccRCC and normal samples. **A.** Up- and down-regulated genes presented in top none biological processes. **B.** Up- and down-regulated genes presented in top eight cellular functions. **C.** Up- and down-regulated genes presented in top eight molecular functions. GO; Gene ontology, ccRCC; Clear cell renal cell carcinoma, and DEGs; Differentially expressed genes.

Using clusterProfiler, GO functional enrichment analysis was performed and the results showed that the upregulated DEGs were significantly enriched in functions like immune system process (GO_BP, P=5.02E^-18^), intracellular (GO_CC, P=5.86 E^-06^) and protein binding (GO_MF, P=1.09E^-18^). Meanwhile, the down-regulated genes were mainly enriched in functions like small molecule metabolic process (GO_BP, P=1.39E^-39^), cytoplasm (GO_CC, P=1.01E^-06^) and binding (GO_MF, P=3.81E^-16^) (Fig .1).

Pathway enrichment analysis showed that the upregulated genes were enriched in pathways like Toll-like receptor signaling pathway (P=1.94E^-05^), while the downregulated genes were enriched in pathways like metabolic pathways (P=1.27E^-28^). The top five enriched pathways are listed in Table 1.

**Table 1:** Top five KEGG pathways enriched by the differentially expressed genes in clear cell renal cell carcinoma


Category	Pathway ID	Pathway	Count	P value

Up	hsa04620	Toll-like receptor signaling pathway	13	1.94E^-05^
	hsa05133	Pertussis	11	2.13E^-05^
	hsa04145	Phagosome	14	3.15E^-04^
	hsa05150	Staphylococcus aureus infection	8	3.18E^-04^
	hsa04666	Fc gamma R-mediated phagocytosis	10	5.19E^-04^
Down	hsa01100	Metabolic pathways	113	1.27E^-28^
	hsa01200	Carbon metabolism	26	9.72E^-16^
	hsa00190	Oxidative phosphorylation	26	5.04E^-14^
	hsa05012	Parkinson’s disease	26	2.56E^-13^
	hsa00280	Valine, leucine and isoleucine degradation	15	1.04E^-11^


P<0.05 was considered to be significantly different. KEGG; Kyoto encyclopedia of genes and genomes.

### Protein-protein interaction network and modules investigation

To dig out more effective information, regarding the DEGs mentioned above, PPI network was constructed on the basis of interaction relationship among the proteins. With score=0.4, a total of 2483 PPI pairs and 643 DEG-encoded proteins were identified. According to the score of degree centrality, TYRO protein tyrosine kinase binding protein (TYROBP, degree=68, up-regulation), cathepsin S (CTSS, degree=53, upregulation), colony stimulating factor 1 receptor (CSF1R, degree=52, up-regulation), Fc fragment of IgE receptor Ig (FCER1G, degree=43, up-regulation), protein tyrosine phosphatase, receptor type C (PTPRC, degree=43, up-regulation), mitogen-activated protein kinase 1 (MAPK1, degree=43, up-regulation), CD53 molecule (CD53, degree=42, up-regulation), Rasrelated C3 botulinum toxin substrate 2 (RAC2, degree=42, up-regulation), cluster of differentiation 14 (CD14, degree=41, up-regulation), cytochrome C, and somatic (CYCS, degree=40, down-regulation) were the top 10 proteins encoded by DEGs.

**Fig 2:**
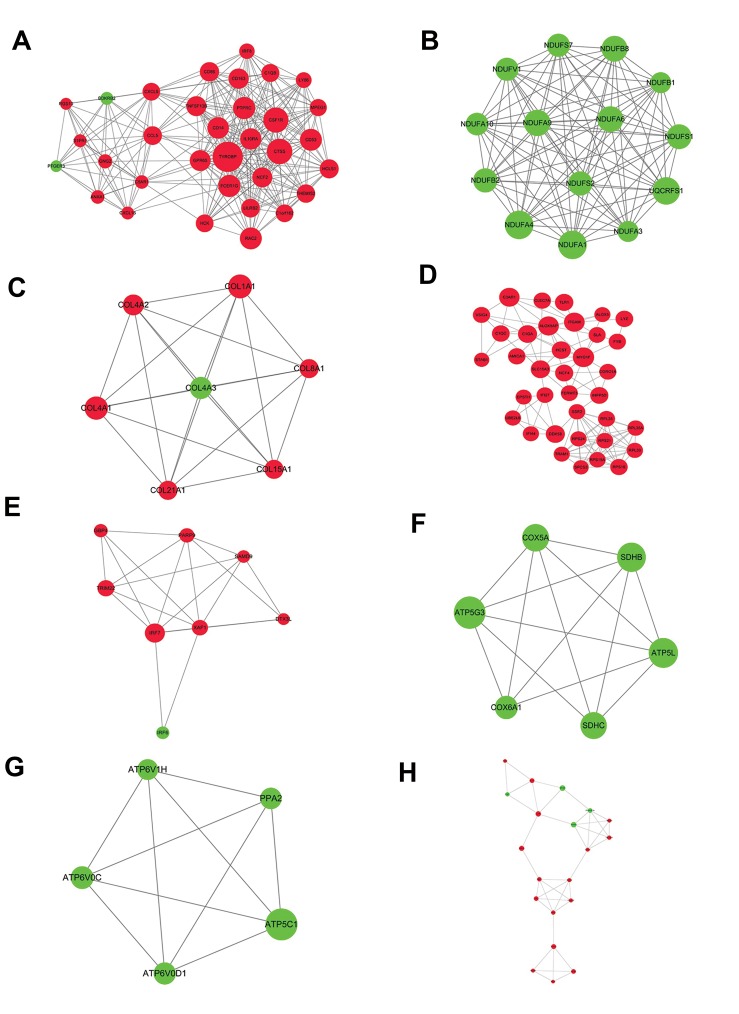
The modules obtained from protein-protein interaction network. **A.** The module “a” was constructed by 33 nodes and 267 interactions. **B.** The module “b” was constructed by 14 nodes and 91 interactions. **C.** The module “c” was constructed by 7 nodes and 21 interactions. **D.** The module “d” was constructed by 36 nodes and 114 interactions. **E.** The module “e” was constructed by 8 nodes and 20 interactions. **F.** The module “f” was constructed by 6 nodes and 14 interactions. **G.** The module “g” was constructed by 5 nodes and 10 interactions. **H.** The module “h” was constructed by 20 nodes and 39 interactions. Green node represents down-regulated gene; red node represents up-regulated gene.

The MOCDE software showed eight modules from PPI network. The detail information was showed in Figure 2. To further investigate crucial pathways involved in the process of ccRCC, the KEGG pathway analysis was performed on DEGs in these modules. Results showed that chemokine signaling pathway (P=4.88E^-05^), Parkinson’s disease (P=3.17E^-24^), protein digestion and absorption (P=3.31E^-08^) and ribosome (P=5.00E^-08^) were the most significant pathways enriched by DEG-encoded proteins in the respectively modules a, b, c and d. Meanwhile, NOTCH signaling pathway (P=3.99E^-02^), oxidative phosphorylation (P=7.89E^-10^), oxidative phosphorylation (P=5.74E^-08^) and adipocytokine signaling pathway (P=2.18E^-02^) were the most significant pathways enriched respectively by modules e, f, g and h. The top two KEGG pathways in each module are listed in Table 2.

### miRNAs-target gene regulatory network analyses

**Fig 3:**
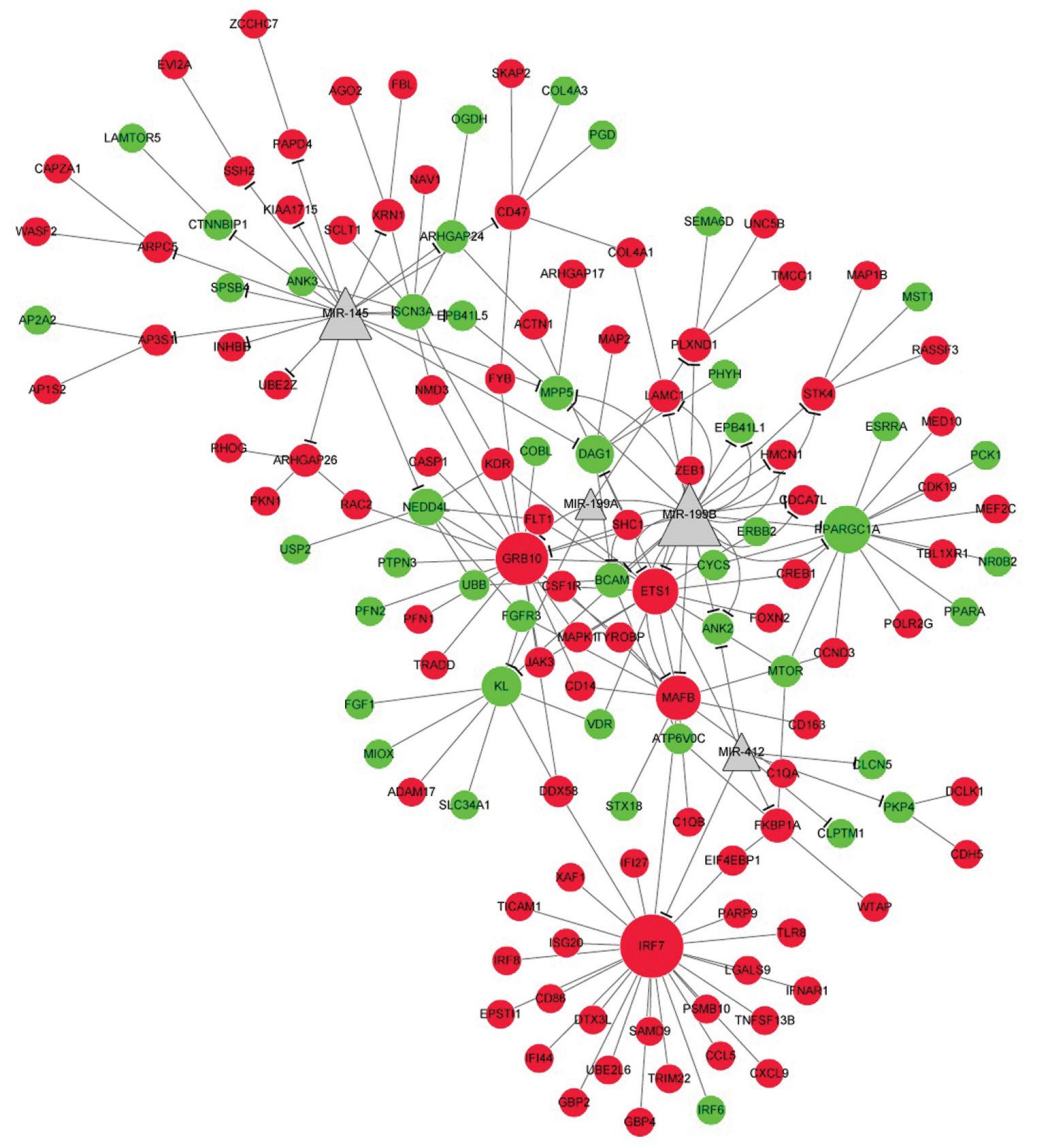
miRNAs-genes regulatory network. Green node represents down-regulated gene, red node represents up-regulated gene and grey triangle represents miRNA.

Here, miRNAs targeted up- and down-regulated genes were investigated based on Enrichr software. Then, the regulatory network was constructed using Cytoscape software. Results showed that there were four miRNAs (including miR-145, miR-199B, miR- 199A and miR-412), 94 up-regulated genes [such as interferon regulatory factor 7 (*IRF7*), TYRO protein tyrosine kinase binding protein (*TYROBP*) and *CD14*)], as well as 45 down-regulated genes [such as PPARG coactivator 1 alpha (*PPARGC1A*), dystroglycan 1 (*DAG1*) and klotho (*KL*)] in the present network (Fig .3).

**Table 2:** Top two KEGG pathways in modules enriched by the differentially expressed genes of clear cell renal cell carcinoma


Module ID	Pathway ID	Pathway name	Count	P value	Genes

a	hsa04062	Chemokine signaling pathway	6	4.88E^-^^0^^5^	CXCL9, RAC2, CXCL16, HCK, GNG2...
	hsa04060	Cytokine-cytokine receptor interaction	6	3.17E^-^^0^^4^	CXCL9, IL10RA, CXCL16, TNFSF13B, CSF1R...
b	hsa05012	Parkinson’s disease	14	3.17E^-^^2^^4^	NDUFB2, NDUFA3, UQCRFS1, NDUFA9, NDUFB8...
	hsa00190	Oxidative phosphorylation	14	3.97E^-^^2^^4^	NDUFB2, NDUFA3, UQCRFS1, NDUFA9, NDUFB8...
c	hsa04974	Protein digestion and absorption	4	3.31E^-^^0^^8^	COL4A2, OL1A1, COL4A1, COL15A1
	hsa04512	ECM-receptor interaction	3	1.15E^-^^0^^5^	COL4A2, COL1A1, COL4A1
d	hsa03010	Ribosome	7	5.00E^-^^0^^8^	RPL35A, RPS24, RPS15A, RPS16, RPL30...
	hsa05150	Staphylococcus aureus infection	4	6.74E^-^^0^^5^	ITGAM, C1QA, C3AR1, C1QC
e	hsa04330	Notch signaling pathway	1	3.99E^-^^0^^2^	DTX3L
	hsa04623	Cytosolic DNA-sensing pathway	1	3.99E^-^^0^^2^	IRF7
f	hsa00190	Oxidative phosphorylation	6	7.89E^-^^1^^0^	SDHB, COX5A, COX6A1, SDHC, ATP5G3...
	hsa05012	Parkinson’s disease	5	9.97E^-^^0^^8^	DHB, COX5A, COX6A1, SDHC, ATP5G3
g	hsa00190	Oxidative phosphorylation	5	5.74E^-^^0^^8^	ATP6V0D1, ATP6V0C, PPA2, ATP5C1, ATP6V1H...
	hsa05110	Vibrio cholerae infection	3	3.94E^-^^0^^5^	ATP6V0D1, ATP6V0C, ATP6V1H
h	hsa04920	Adipocytokine signaling pathway	3	2.18E^-^^0^^2^	MTOR, PPARGC1A, PPARA


KEGG; Kyoto Encyclopedia of Genes and Genomes.

## Discussion

ccRCC is one of the most common RCC identified in adults presenting the worst prognosis among the common epithelial tumors of kidney. In this study, total 955 DEGs were identified in ccRCC from normal samples. GO analysis showed that these DEGs, including up-regulated *TYROBP* and *IRF7*, and down-regulated *PPARGC1A*, were mainly enriched in functions like immune system and small molecule metabolic processes. KEGG enrichment analysis demonstrated that these DEGs were significantly enriched in the pathways like Toll-like receptor signaling pathway as well as metabolic pathways. Furthermore, *IRF7* was predicted to be targeted by miR-412 and *PPARGCA1* could be targeted by miR-199B.

Immune system protects host body against disease. Aberration of immune system could lead to inflammatory diseases, autoimmune diseases and cancer (17). Importantly, ccRCC has shown extensive responses to immune checkpoint blockade therapies (18). Based on an in-depth immune profiling study, Chevrier et al. (19) identified that *CD38* and *CD204* involved in the tumor microenvironment modulate cancer progression. Previous study has presented that orchestration of immune checkpoints has prognostic value in ccRCC (20), but detailed mechanism of the checkpoints in ccRCC is yet unclear.

*TYROBP* is the encoding gene of a transmembrane signaling polypeptide, which possesses an immunoreceptor tyrosine-based activation motif in its cytoplasmic domain (21). Previous study reported that TYROBP associated with the killer cell immunoglobulin-like receptor family and it served as an activating signal transduction element in cells (22). *TYROBP* is reported to be related to Alzheimer’s pathology (21). Deficiency of *TYROBP* is neuroprotective in a mouse model with early Alzheimer’s pathology. Meanwhile, the bone remodeling and brain function also depend on the integrity of *TYROBP* signal (23). In addition, it has been documented that TYROBP presents a general function in inflammatory responses in microglia via the Jun NH2-terminal kinase (JNK) signaling pathway (24). However, very rare studies focus on *TYROBP* correlation with ccRCC. In the current study, functional enrichment analysis showed that *TYROBP* was significantly enriched in the BP of immune system, indicating that *TYROBP* might also play critical role in the pathogenic inflammatory response of ccRCC. However, further analysis of *TYROBP* is still required to reveal the exact mechanism involved in pathogenesis of ccRCC.

*IRF7*, encoded interferon regulatory factor, is a member of type I interferon regulatory transcription factor family, which plays a critical role in the innate immunity (25). *IRF7* silencing promotes bone metastasis of breast cancer via immune escape way (26). Coit et al. (27) have documented that *IRF7* plays critical role in renal involved in lupus via regulating demethylation of DNA in naive CD4^+^ T cells. It was also reported that *IRF7* can serve as a therapeutic target against renal tissue damage caused by bacterial infection (28). In the current study, functional enrichment analysis revealed that *IRF7* was significantly enriched in the Toll-like receptor signaling pathway and immune response biological process. Toll-like receptor signaling pathway has been reported to play crucial roles in the inflammation, infection and cancer (29). Moreover, IRF7 was predicted to be targeted by miR-412 in ccRCC. miR-412 is a mature type of miR-142, contributing to important functions in inflammatory and immune response among different diseases (30). Taken together, we speculated that miR-412 might present a critical role in the immune response of ccRCC via targeting IRF7 in Toll-like receptor signaling pathway.

*PPARGCA1* is the encoding gene of PPARG coactivator 1α, which is a co-regulator of mitochondrial biogenesis and oxidative phosphorylation. Cho et al. (31) have documented that genetic variation of *PPARGCA1* regulates diet-associated inflammation in colorectal cancer. Moreover, polymorphisms of *PPARGCA1* and lower expression of *PPARGCA1* are risk factors in the pathogenesis of non-alcoholic fatty liver disease, characterized by abnormal inflammatory response during etiology (32). In the current study, down-regulation of *PPARGCA1* was identified in ccRCC samples and functional enrichment showed that *PPARGCA1* was significantly enriched in the immune response biological process. Further analysis showed that *RRARGCA1* was regulated by miR-199b. miR-199b is down-regulated in several solid tumors (33). Hou et al. (34) have documented that down-regulation of miR-199b strongly correlated with poor survival of HCC patients. In addition, miR-199b associated with poor survival of ovarian cancer patients, and loss of miR-199b results in hypermethylation in ovarian cancer (35). These evidences indicated that miR- 199b might contribute to the pathogenesis of ccRCC via regulating *PPARGCA1* expression.

## Conclusion

Abnormal immune response might be an important mechanism of ccRCC. *TYROBP, IRF7* and *PPARGCA1* might play important roles in ccRCC via taking part in the immune system. Furthermore, miR-199B and miR-412 might serve critical roles in the regulation of immune response via targeting *PPRARGCA1* and *IRF7*, respectively. Considering these findings, miR-199b and miR-412 might be used as the prognostic target for ccRCC gene therapy.

## Supplementary PDF


