# Supplementary material for: Gene Expression Microarray Data Meta-Analysis Identifies Candidate Genes and Molecular Mechanism Associated with Clear Cell Renal Cell Carcinoma
Source: Cell J. 2019 Dec 15;22(3):386–93. doi: 10.22074/cellj.2020.6561 (PMC6947001; doi:10.22074/cellj.2020.6561)
Supplement: Supplementary file 1 [file Cell-J-22-386-s01.pdf]

CELL JOURNAL (Yakhteh)

# Supplementary Information for

# Gene Expression Microarray Data Meta-Analysis Identifies Candidate Genes and Molecular Mechanism Associated with Clear Cell renal Cell Carcinoma

Ying Wang, Ph.D.[1#*], Haibin Wei, M.Sc.[2#], Lizhi Song, M.Sc.[1], Lu Xu, M.Sc.[1], Jingyao Bao, B.Sc.[1], Jiang Liu, Ph.D.[1*]

1. Institute of Aging Research, School of Medicine, Hangzhou Normal University, Hangzhou, Zhejiang, China
2. Department of Pathology, Zhejiang Cancer Hospital, Hangzhou, Zhejiang, China

#The first two authors equally contributed to this work.

*Corresponding Address: Institute of Aging Research, School of Medicine, Hangzhou Normal University, Hangzhou, Zhejiang, China
Emails: flashingdancer@163.com, Jennings_L143@126.com

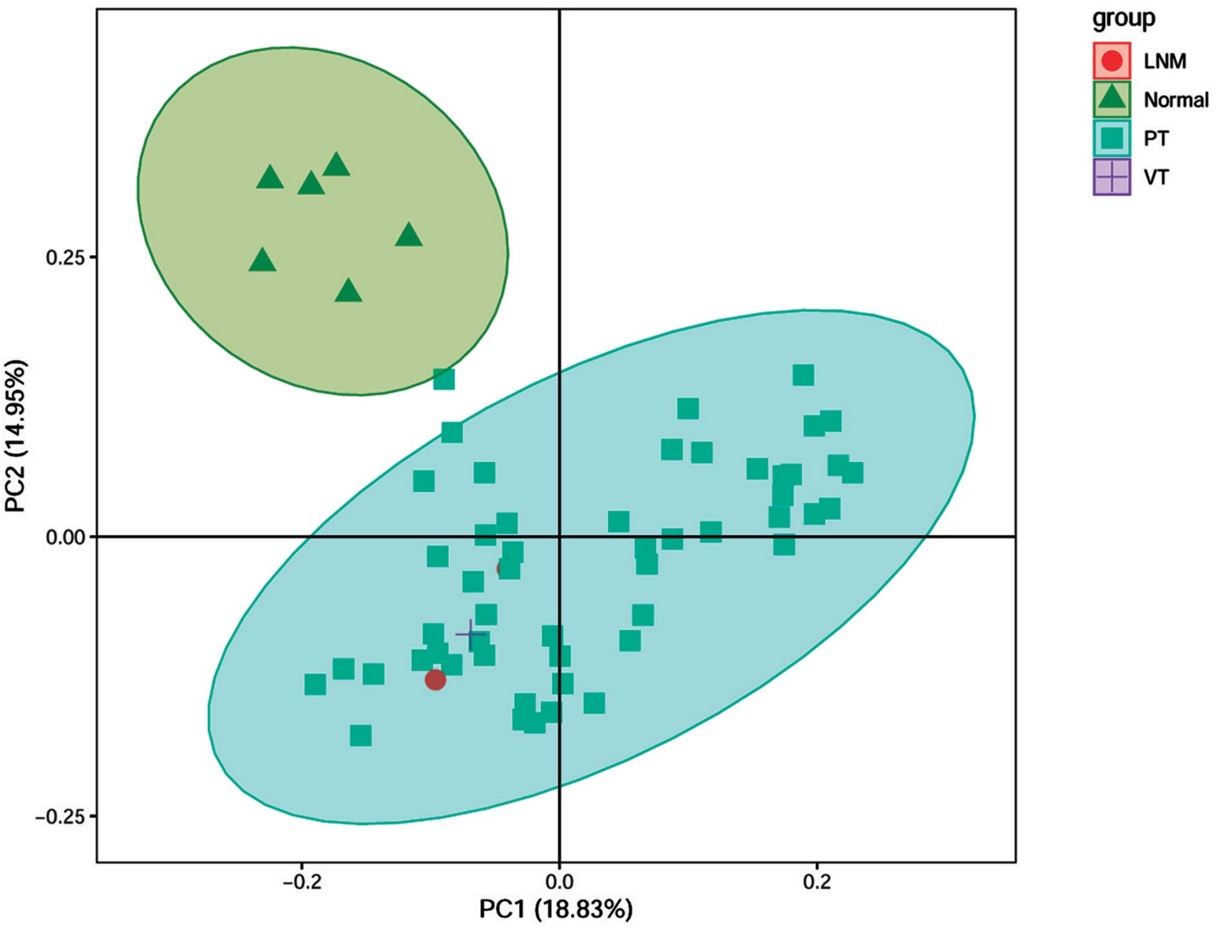


**Fig.S1:** Principal component analysis for 56 clear cell renal cell carcinoma tissue samples and 6 normal tissue samples. LNM; Lymph node metastasis, VT; Venous thrombus metastasis, PT; Primary tumor.

**Table S1:** The samples selected from GSE781

| Accession | Sample | Age (Y) | Gender | Kidney | Grade | Capsule penetration | Sinus invasion |
|---|---|---|---|---|---|---|---|
| GSM11805 | N035 normal human kidney U133A | 70 | F | right | III | + | - |
| GSM11814 | C035 renal clear cell carcinoma U133A | 70 | F | right | III | + | - |
| GSM11823 | N023 normal human kidney U133A | 72 | F | right | I | + | - |
| GSM11830 | C023 renal clear cell carcinoma U133A | 72 | F | right | I | + | - |
| GSM12067 | C001 renal clear cell carcinoma U133A | 58 | M | left | I | + | - |
| GSM12075 | N001 normal human kidney U133A | 58 | M | left | I | + | - |
| GSM12079 | C005 renal clear cell carcinoma U133A | 64 | M | right | I | - | - |
| GSM12098 | N005 normal human kidney U133A | 64 | M | right | I | - | - |
| GSM12100 | C011 renal clear cell carcinoma U133A | 55 | M | left | III | + | - |
| GSM12105 | C032 renal clear cell carcinoma U133A | 65 | F | right | III | + | + |
| GSM12268 | N1 normal human kidney U133A | 51 | M | left | III | - | + |
| GSM12270 | C2 renal clear cell carcinoma U133A | 67 | M | right | II | - | + |
| GSM12283 | N2 renal clear cell carcinoma U133A | 67 | M | right | II | - | + |
| GSM12298 | C3 renal clear cell carcinoma U133A | 50 | M | left | II | - | - |
| GSM12300 | N3 renal clear cell carcinoma U133A | 50 | M | left | II | - | - |
| GSM12399 | C4 renal clear cell carcinoma U133A | 65 | M | left | I | + | - |
| GSM12444 | N4 renal clear cell carcinoma U133A | 65 | M | left | I | + | - |

F; Female and M; Male.

**Table S2:** The samples selected from GSE6344

| Accession | Sample | Type | Stage | Genechips |
|---|---|---|---|---|
| GSM146778 | PT#2 | Normal | I | HG-U133A |
| GSM146779 | PT#2 | Tumor | I | HG-U134A |
| GSM146780 | PT#3 | Normal | I | HG-U135A |
| GSM146781 | PT#3 | Tumor | I | HG-U136A |
| GSM146782 | PT#4 | Normal | I | HG-U137A |
| GSM146783 | PT#4 | Tumor | I | HG-U138A |
| GSM146784 | PT#5 | Normal | I | HG-U139A |
| GSM146785 | PT#5 | Tumor | I | HG-U140A |
| GSM146786 | PT#6 | Normal | I | HG-U141A |
| GSM146787 | PT#6 | Tumor | I | HG-U142A |
| GSM146788 | PT#8 | Tumor | II | HG-U143A |
| GSM146789 | PT#8 | Normal | II | HG-U144A |
| GSM146790 | PT#9 | Normal | II | HG-U145A |
| GSM146791 | PT#9 | Tumor | II | HG-U146A |
| GSM146792 | PT#10 | Normal | II | HG-U147A |
| GSM146793 | PT#10 | Tumor | II | HG-U148A |
| GSM146794 | PT#11 | Normal | II | HG-U149A |
| GSM146795 | PT#11 | Tumor | II | HG-U150A |
| GSM146796 | PT#12 | Normal | II | HG-U151A |
| GSM146797 | PT#12 | Tumor | II | HG-U152A |

**Table S3:** The samples selected from GSE53000

| Accession | Sample | Type | Tissue | Age (Y) | Sex |
|---|---|---|---|---|---|
| GSM1279951 | EV002 | Normal kidney | Normal adult kidney | 59 | M |
| GSM1279952 | EV003 | Normal kidney | Normal adult kidney | 64 | F |
| GSM1279953 | EV005 | Normal kidney | Normal adult kidney | 79 | F |
| GSM1279954 | EV007 | Normal kidney | Normal adult kidney | 59 | F |
| GSM1279955 | RMH002 | Normal kidney | Normal adult kidney | 63 | M |
| GSM1279956 | RMH008 | Normal kidney | Normal adult kidney | 63 | M |
| GSM1279957 | EV002 | Primary tumor region | Clear cell renal cell carcinoma | 59 | M |
| GSM1279959 | EV002 | Primary tumor region | Clear cell renal cell carcinoma | 59 | M |
| GSM1279960 | EV002 | Primary tumor region | Clear cell renal cell carcinoma | 59 | M |
| GSM1279962 | EV002 | Primary tumor region | Clear cell renal cell carcinoma | 59 | M |
| GSM1279963 | EV002 | Primary tumor region | Clear cell renal cell carcinoma | 59 | M |
| GSM1279964 | EV003 | Primary tumor region | Clear cell renal cell carcinoma | 64 | F |
| GSM1279965 | EV003 | Primary tumor region | Clear cell renal cell carcinoma | 64 | F |
| GSM1279966 | EV003 | Primary tumor region | Clear cell renal cell carcinoma | 64 | F |
| GSM1279967 | EV003 | Primary tumor region | Clear cell renal cell carcinoma | 64 | F |
| GSM1279968 | EV003 | Primary tumor region | Clear cell renal cell carcinoma | 64 | F |
| GSM1279969 | EV005 | Primary tumor region | Clear cell renal cell carcinoma | 79 | F |
| GSM1279970 | EV005 | Primary tumor region | Clear cell renal cell carcinoma | 79 | F |
| GSM1279971 | EV005 | Primary tumor region | Clear cell renal cell carcinoma | 79 | F |
| GSM1279972 | EV005 | Primary tumor region | Clear cell renal cell carcinoma | 79 | F |
| GSM1279973 | EV005 | Primary tumor region | Clear cell renal cell carcinoma | 79 | F |
| GSM1279974 | EV005 | Primary tumor region | Clear cell renal cell carcinoma | 79 | F |
| GSM1279975 | EV005 | Primary tumor region | Clear cell renal cell carcinoma | 79 | F |
| GSM1279976 | EV006 | Primary tumor region | Clear cell renal cell carcinoma | 65 | M |
| GSM1279977 | EV006 | Primary tumor region | Clear cell renal cell carcinoma | 65 | M |
| GSM1279978 | EV006 | Primary tumor region | Clear cell renal cell carcinoma | 65 | M |
| GSM1279979 | EV006 | Primary tumor region | Clear cell renal cell carcinoma | 65 | M |
| GSM1279980 | EV006 | Primary tumor region | Clear cell renal cell carcinoma | 65 | M |
| GSM1279982 | EV006 | Primary tumor region | Clear cell renal cell carcinoma lymph node metastasis | 65 | M |
| GSM1279983 | EV006 | Lymph node metastasis | Clear cell renal cell carcinoma lymph node metastasis | 65 | M |
| GSM1279984 | EV006 | Lymph node metastasis | Clear cell renal cell carcinoma | 65 | M |
| GSM1279985 | EV007 | Primary tumor region | Clear cell renal cell carcinoma | 59 | F |
| GSM1279986 | EV007 | Primary tumor region | Clear cell renal cell carcinoma | 59 | F |

**Table S3:** Continued

| Accession | Sample | Type | Tissue | Age (Y) | Sex |
|---|---|---|---|---|---|
| GSM1279987 | EV007 | Primary tumor region | Clear cell renal cell carcinoma | 59 | F |
| GSM1279988 | EV007 | Primary tumor region | Clear cell renal cell carcinoma | 59 | F |
| GSM1279989 | EV007 | Primary tumor region | Clear cell renal cell carcinoma | 59 | F |
| GSM1279990 | EV007 | Primary tumor region | Clear cell renal cell carcinoma | 59 | F |
| GSM1279991 | EV007 | Primary tumor region | Clear cell renal cell carcinoma | 59 | F |
| GSM1279992 | RK26 | Primary tumor region | Clear cell renal cell carcinoma | 47 | M |
| GSM1279993 | RK26 | Primary tumor region | Clear cell renal cell carcinoma | 47 | M |
| GSM1279994 | RK26 | Primary tumor region | Clear cell renal cell carcinoma | 47 | M |
| GSM1279995 | RK26 | Primary tumor region | Clear cell renal cell carcinoma | 47 | M |
| GSM1279996 | RK26 | Primary tumor region | Clear cell renal cell carcinoma | 47 | M |
| GSM1279997 | RK26 | Primary tumor region | Clear cell renal cell carcinoma | 47 | M |
| GSM1279998 | RK26 | Primary tumor region | Clear cell renal cell carcinoma | 47 | M |
| GSM1279999 | RK26 | Primary tumor region | Clear cell renal cell carcinoma | 47 | M |
| GSM1280000 | RK26 | Primary tumor region | Clear cell renal cell carcinoma | 47 | M |
| GSM1280001 | RMH002 | Primary tumor region | Clear cell renal cell carcinoma | 63 | M |
| GSM1280002 | RMH002 | Primary tumor region | Clear cell renal cell carcinoma | 63 | M |
| GSM1280003 | RMH002 | Primary tumor region | Clear cell renal cell carcinoma | 63 | M |
| GSM1280004 | RMH002 | Primary tumor region | Clear cell renal cell carcinoma | 63 | M |
| GSM1280005 | RMH004 | Primary tumor region | Clear cell renal cell carcinoma | 61 | M |
| GSM1280006 | RMH004 | Primary tumor region | Clear cell renal cell carcinoma | 61 | M |
| GSM1280007 | RMH004 | Primary tumor region | Clear cell renal cell carcinoma | 61 | M |
| GSM1280010 | RMH004 | Primary tumor region | Clear cell renal cell carcinoma | 61 | M |
| GSM1280012 | RMH004 | Venous thrombus | Clear cell renal cell carcinoma venous thrombus | 61 | M |
| GSM1280013 | RMH008 | Primary tumor region | Clear cell renal cell carcinoma | 63 | M |
| GSM1280014 | RMH008 | Primary tumor region | Clear cell renal cell carcinoma | 63 | M |
| GSM1280015 | RMH008 | Primary tumor region | Clear cell renal cell carcinoma | 63 | M |
| GSM1280016 | RMH008 | Primary tumor region | Clear cell renal cell carcinoma | 63 | M |
| GSM1280017 | RMH008 | Primary tumor region | Clear cell renal cell carcinoma | 63 | M |
| GSM1280018 | RMH008 | Primary tumor region | Clear cell renal cell carcinoma | 63 | M |

F; Female and M; Male.
